# Multisensory Perceptual Learning of Temporal Order: Audiovisual Learning Transfers to Vision but Not Audition

**DOI:** 10.1371/journal.pone.0011283

**Published:** 2010-06-23

**Authors:** David Alais, John Cass

**Affiliations:** 1 School of Psychology, University of Sydney, Sydney, Australia; 2 School of Psychology, University of Western Sydney, Milperra, Australia; 3 MARCS Auditory Laboratories, University of Western Sydney, Milperra, Australia; L'université Pierre et Marie Curie, France

## Abstract

**Background:**

An outstanding question in sensory neuroscience is whether the perceived timing of events is mediated by a central supra-modal timing mechanism, or multiple modality-specific systems. We use a perceptual learning paradigm to address this question.

**Methodology/Principal Findings:**

Three groups were trained daily for 10 sessions on an auditory, a visual or a combined audiovisual temporal order judgment (TOJ). Groups were pre-tested on a range TOJ tasks within and between their group modality prior to learning so that transfer of any learning from the trained task could be measured by post-testing other tasks. Robust TOJ learning (reduced temporal order discrimination thresholds) occurred for all groups, although auditory learning (dichotic 500/2000 Hz tones) was slightly weaker than visual learning (lateralised grating patches). Crossmodal TOJs also displayed robust learning. Post-testing revealed that improvements in temporal resolution acquired during visual learning transferred within modality to other retinotopic locations and orientations, but not to auditory or crossmodal tasks. Auditory learning did not transfer to visual or crossmodal tasks, and neither did it transfer within audition to another frequency pair. In an interesting asymmetry, crossmodal learning transferred to all visual tasks but not to auditory tasks. Finally, in all conditions, learning to make TOJs for stimulus onsets did not transfer at all to discriminating temporal offsets. These data present a complex picture of timing processes.

**Conclusions/Significance:**

The lack of transfer between unimodal groups indicates no central supramodal timing process for this task; however, the audiovisual-to-visual transfer cannot be explained without some form of sensory interaction. We propose that auditory learning occurred in frequency-tuned processes in the periphery, precluding interactions with more central visual and audiovisual timing processes. Functionally the patterns of featural transfer suggest that perceptual learning of temporal order may be optimised to object-centered rather than viewer-centered constraints.

## Introduction

Temporal processes are an essential aspect of perception and action. The brain needs be sensitive to timing on a variety of scales to ensure our survival. On the briefest scale, temporal differences on a microsecond scale are used a cue to localise auditory sound sources, while many orders of magnitude longer are the approximately 24-hour circadian rhythms that govern appetite and the sleep/wake cycle. Towards the shorter end of these two extremes is a very critical time band that ranges from 10 s to 100 s of milliseconds [1]. This sub-second range, the focus of the current paper, is essential for many important sensory and perceptual tasks including speech perception, motion perception, motor coordination [2], [3], [4]. Sub-second timing is also essential for coordinating crossmodal interactions and multisensory integration [5], [6].

When compared to what is known about spatial perception, time perception is poorly understood. Many key aspects of the neural bases of time perception remain unclear. One continuing debate is whether there is a single central clock governing time perception or whether multiple peripheral clocks exist [7], [8], [9], [10]. If timing is governed by a central mechanism, it is likely to be a supramodal process subserving timing of events regardless of modality of origin. However, there may instead be multiple peripheral clocks, with one existing for each sensory modality. Further, it is possible that independent clocks may exist within sensory modalities, perhaps one for each feature, attribute or location. Regardless of the ‘central vs peripheral’ question, it is quite possible that multiple clocks would be needed to cover the vast range of time scales that must be encoded. There are around 10 orders of magnitude from the microsecond scale used in audition to the day-long circadian cycle and it is unlikely that one type of clock could serve for all time scales [11].

One standard approach to investigating sub-second time perception has been to use temporal interval discrimination [12], [13], [14], [15]. In this paradigm, a brief marker stimulus is used to indicate the beginning and end of a time period. Temporal discrimination thresholds are generally measured in a two-interval procedure by having observers indicate whether a standard interval is shorter or longer than a second comparison stimulus. In this way, a discrimination threshold can be obtained to measure sensitivity to a particular time period. Studies along these lines have shown that perception of interval duration follows Webers's law in that increment thresholds are about 10–15% of the standard interval, and that this holds over a wide range of temporal intervals [16], [17], [18].

Thresholds for temporal interval discrimination, as for any perceptual threshold, may generally be improved by extended practice over a number of days. This is known as perceptual learning. By definition, perceptual learning is simply a relatively permanent improvement (e.g., over weeks or months) in perceptual acuity as a result of consistent practice [19]. On virtually any perceptual task, daily practice will improve thresholds so that subjects effectively improve their perceptual acuity. This can be shown for tasks as simple as orientation discrimination and contrast detection in vision [20], [21], where after about 8 to 10 days of practice, discrimination thresholds descend asymptotically to a new lower baseline. In audition, several studies have demonstrated that perceptual learning occurs in auditory interval and temporal order discrimination [8], [22], [23], [24]. More recently, a number of studies have examined perceptual learning in multisensory contexts [25], [26]. All these studies reflect the surprising degree of plasticity in the adult brain [27], [28], [29].

Once perceptual learning has taken place, one of the key questions is the extent to which it may or may not transfer to other tasks. If two different perceptual tasks utilise the same neural process, then improved performance on one task due to perceptual learning should lead to improved performance on the other. To demonstrate this, performance on the second task is measured prior to the commencement of the daily training sessions and then again after the final session. If performance on the second task is found to have improved without exposure to the intervening training sessions, then the perceptual learning benefit has generalised to the second task, implying that they are subserved by common processes. If the two tasks use distinct process, then no transfer of learning would be expected. The test of learning generalisation then provides a potentially powerful method for revealing whether neural timing processes are central or not, or indeed whether there are multiple processes within a modality. Clearly, if there were a single, central timing process, then it would be supramodal and therefore any gains in temporal resolution due to perceptual learning in the auditory domain should transfer to the visual domain (and *vice versa*).

In this paper, we will use the perceptual learning paradigm and tests of learning generalisation to determine whether commonalities exist between auditory, visual, and audiovisual timing processes. Separate groups of subjects will be trained to make temporal order judgments (TOJs) with visual stimuli, auditory stimuli, and audiovisual stimuli. By testing for transfer of learned improvements in temporal discrimination from audition to vision (and *vice versa*) and from unimodal to bimodal (and *vice versa*) we will obtain data that bears closely on the question of whether timing is central and supramodal, or peripheral and modality based. A previous study using temporal interval discrimination (as described above) measured transfer of learning from audition and vision and found significant transfer, although this was limited to the trained interval duration [23]. Our motive for returning to this question is twofold. First, we wanted to test transfer of learned timing improvements in a more complete design. Wright et al ran an auditory group, with a post-training test for visual timing, but did not run a visual learning vision group, or an audiovisual group. Second, we chose to use TOJs instead of temporal interval discrimination.

The choice of TOJs instead of interval discrimination because it offers several advantages. First, there is a potentially confounding memory element in the interval discrimination paradigm. Because there are two stimulus presentations in interval discrimination (one the standard, the other the comparison stimulus) followed by a choice as to which was longer, the former must be retained in memory and the final decision is made by referring back to memory. This allows potential problems due to memory encoding or retrieval to intervene in the measurement, potentially contaminating temporal data with memory effects. Second, it may well be that the actual interval in an interval discrimination task is modality free since it is simply a period of time bounded by a brief marker stimulus (for example, a brief beep to indicate the start and end of the period). It is not clear that simply changing the modality of the marker stimulus (e.g., from a brief beep to a flash of light) effectively changes the modality of the bounded interval from auditory to visual. That is, the elapsed time between the marker stimuli may well be a modality-free duration. In this were so, the ‘crossmodal’ transfer of learning observed in Wright, *et al*. 's experiment [23] would be entirely as expected since changing the markers would leave the fundamental nature of the temporal interval unchanged. A third advantage afforded by TOJs over interval discrimination tasks is that no arbitrary choices about interval duration need be made. In the TOJ task, the stimulus onset asynchrony is simply reduced with an adaptive staircase to find the Δ*t* threshold for discrimination. The TOJ method therefore goes straight to the heart of the sub-second temporal limit in sensory processing that is of interest in this experiment.

By using TOJs in visual, auditory and audiovisual learning groups and testing for transfer of learning back to the other modality (or bimodality), we hope to learn more about whether the processes underlying sub-second time perception are central and supramodal, or peripheral and modality specific.

## Results

**Figure 1:**
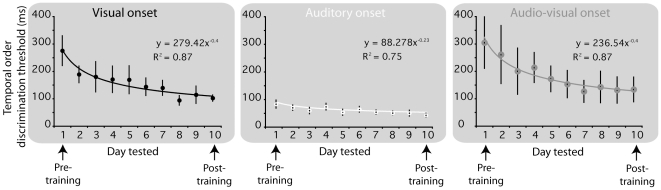
Temporal order discrimination thresholds measured in each of three sensory modalities (visual, auditory and audio-visual) across 10 separate days. Data points show group means and error bars show ±1 standard error of the mean.

Figure 1 plots temporal order onset discrimination thresholds across the ten days of training (including pre- and post-training), with data for the auditory, visual and audiovisual groups shown separately. The first point is that robust learning occurred in all groups, with the improvement in TOJ thresholds well described by the characteristic negative power function for all groups. The rate of improvement in TOJ thresholds was similar for the visual and audiovisual groups (exponents of −0.4), although improvement for the auditory group occurred at a much slower rate (exponent of −.23). The second point to note is that the pre-training baselines (thresholds for day one) differed significantly across the three groups. Prior to any training on temporal discrimination, thresholds were far better for audition (group mean = 82 ms) than for vision (group mean = 275 ms), and audiovisual temporal discrimination was worse than both (group mean = 305 ms). Thus, although learning occurred at the slowest rate in audition, baseline temporal discrimination was by far the best in this group. From these baselines, even with the faster rate of improvement in the visual and audiovisual groups, the same group order was observed on day 10: audition (45 ms) better than vision (102 ms), and vision better than audiovision (134 ms). It is also clear that 10 days of training was largely sufficient to stabilise TOJ onset thresholds at a lower asymptotic level of performance as the points for the last three training sessions in each group show no tendency to continue to decline.

**Figure 2:**
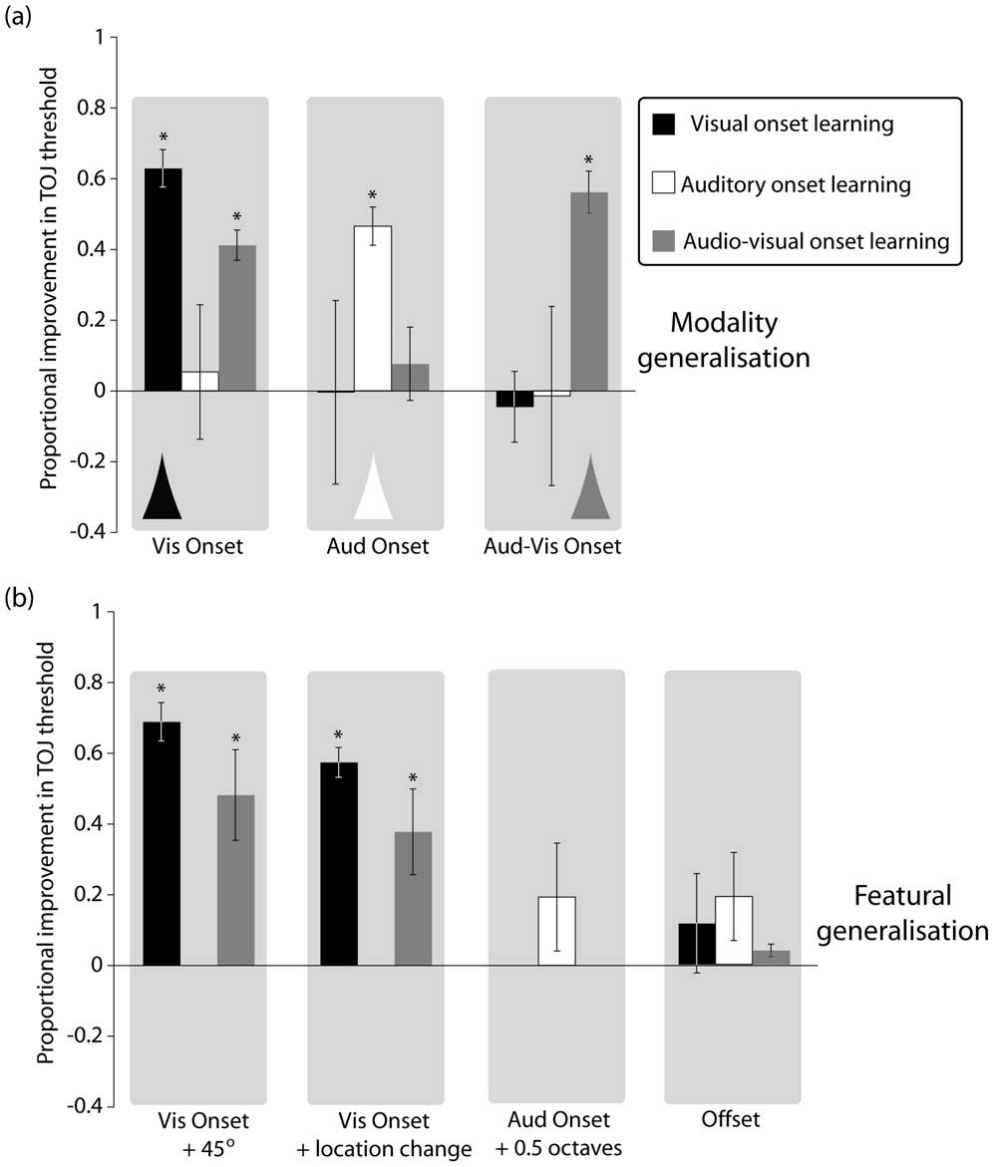
Proportional improvements in TOJ threshold performance measured for the various stimulus modalities and features as a consequence of different intervening training tasks. The bars plot group means and error bars show ±1 standard error of the mean. Black bars represent TOJ improvement following visual onset training, white bars, auditory onset training, and grey bars, audio-visual onset training. Asterisks indicate significant threshold improvement (α<.05). (a) Comparison of the generalisability of onset learning within and between stimulus modalities. Triangles signify within-modality improvement. Note that the only instance of between-modality improvement occurred for visual onset tasks following audio-visual onset training. (b) Comparison of the generalisabilty of onset learning to other stimulus features. Whereas visual and audio-visual learning generalised across both orientation and location to visual onset judgments, auditory learning failed to generalise to other frequencies. Note also the lack of generalisation from onsets to offsets.

Once the eight days of training on TOJ onset discrimination were completed, we ran a battery of final post-training conditions to test for generalisation of perceptual learning to other training conditions. Three types of generalisation were tested: (i) transfer of onset TOJ learning in one modality to the other modality or to the bimodal condition (Figure 2a); (ii) transfer of onset TOJ learning to offset TOJs; (iii) transfer of onset TOJ learning within a modality from one feature to another feature (Figure 2b). Figure 2a shows how learning of onset TOJs generalises between sensory modalities. Each panel plots the proportionate change in onset TOJ performance from pre- to post-training (i.e., from day 1 (pre-training) to day 10 (post-training)) following training in a particular sensory modality, either visual, auditory, or audiovisual. Proportionate changes in TOJ thresholds from pre-test to post-test were calculated by: 1- (threshold_post_/threshold_pre_). The histogram marked with an arrow in each panel indicates the case where the training modality matches the pre- and post-training modalities. The other histograms (without arrows) in Figure 2a represent cases where the pre- and post-training was done in one modality and the intervening training was carried out in another modality (as coded by grey level (black = visual onset learning; white = auditory onset learning; grey = audio-visual onset learning). Any improvement, therefore, that is significantly greater than zero in these non-arrowed columns indicates a transfer of TOJ onset learning from training in another modality. The only condition where this applies is in the first panel: training on audiovisual onset TOJs led to better performance on discriminating the order of visual onsets (purple column), although curiously, audiovisual training did not transfer to auditory onset discrimination (second panel). TOJ training on visual stimuli or on auditory stimuli did not exhibit any transfer.

Figure 2b shows the tests of generalisation within modalities to other features. In the first two panels, it can be seen that visual training on horizontal (target)/vertical (pedestal) grating patches (black columns) led to significant TOJ improvements in post-training tests on obliquely tilted (orthogonally oriented target/pedestal) grating patches (panel 1) and on horizontal/vertical grating patches at another retinal location (panel 2). Visual learning, therefore, generalised across feature change and location change. The first two panels also show that audiovisual training (grey columns) involving a vertical grating patch as the visual component also led to significant TOJ improvements in vision-only post-training. This occurred on obliquely tilted grating patches (panel 1) and on vertical grating patches at another retinal location (panel 2). The third panel shows an absence of generalisation of learning within the auditory modality. Auditory TOJ training on dichotic tones of 500 (pedestal) and 2000 Hz (target) did not produce a significant improvement in auditory TOJ threshold on tones of 1500 and 3000 Hz.

Finally, the data in the rightmost panel of Figure 2b are for the test of learning generalisation from onset discrimination to offset discrimination. Aside from the initial pre-test phase (day 1) all other training involved discriminating the temporal order of stimulus onsets. While this led to strong improvements in temporal acuity for stimulus onsets (Figure 1), the right panel shows that there is no significant improvement in thresholds for discriminating stimulus offsets, even though the offset post-training stimuli were in the same modality and involved no feature change. It is clear, then, that improvements in temporal discrimination gained from training on stimulus onsets do not transfer to stimulus offsets.

## Discussion

The original motive for this study was to use a perceptual learning paradigm to investigate whether timing in the sub-second range is central and supramodal, or peripheral and modality specific. The results do not satisfy either alternative and instead point to a more complex picture. On one hand, the unimodal data are very clear. They show that the improvement in temporal resolution for onset TOJs following unimodal training did not transfer at all to the other unimodal condition–neither from vision to audition, nor from audition to vision. In the absence of any further data, this would seem a strong case against a central supramodal mechanism mediating sub-second TOJs and instead favour a model of separate timing mechanisms within each modality. This is the same pattern reported in a very recent multisensory study on perceptual learning of asynchrony-detection which supports the same conclusion [30]. However, there are two key aspects of the data that rule out both of these simplified alternatives. The first of these concerns the two tests of generalisation following audiovisual training back to unimodal visual and auditory tasks. The substantial improvement in temporal acuity following audiovisual training transferred fully to the visual task and did so regardless of variations in visual feature and stimulus location. Yet, bimodal-to-unimodal transfer of learning, which would be strongly indicative of a single central clock, was asymmetrical in that there was no transfer of audiovisual learning to audition–not even for the auditory frequency used during audiovisual training. The second aspect of the data which complicates the interpretation concerns transfer of learning within modalities. Although visual learning transferred across feature space to other orientations and locations, consistent with a single clock for vision, auditory learning was very specific to the trained stimulus and showed no transfer to tones of another frequency. In contrast to the visual data, this specificity of auditory learning implies independent clocks for separate auditory features.

Despite the evidence for modality specificity in auditory timing, there are two arguments which support the existence of a supramodal timer. First, there was clear evidence of learning on the audiovisual task, suggesting there is supramodal timer whose performance can be improved by training. Second, such a timer appears not simply to receive trained unimodal signals as its inputs as there was no transfer of auditory learning nor of visual learning to the audiovisual task. This second result suggests an important point: the audiovisual clock appears not to receive unimodal signals fed-forward from modality-specific clocks within a single timing network. Clearly, an audiovisual clock receiving timing information from peripheral unimodal clocks would inherit more precise timing signals following unimodal training, in which case unimodal-to-bimodal transfer of learning should have occurred. This result did not occur, suggesting that the audiovisual TOJs are mediated by a separate timing network. The data also suggest another important conclusion: the absence of unimodal-to-bimodal transfer also excludes the possibility that the audiovisual task was done at a post-perceptual cognitive level based on a comparsion of signals arriving from the auditory and visual streams. Had this been the case, more precise unimodal signals would have permitted better onset discrimination bimodally. Together, the data point to a bona fide supramodal timing network, but one that exists independently of other modality specific timers.

The one complication to this interpretation is that learned improvements in making audiovisual TOJs did transfer to uimodal visual TOJs, indicating there is not a complete independence. Why would audiovisual learning transfer to unimodal visual tasks but not to auditory tasks? One approach to multisensory integration that might inform an answer focuses on the relative reliability of sensory components. On this view, when redundant stimulus cues are combined, perceptual decisions are mainly determined by the more reliable (i.e., perceptually precise) component [31], [32], [33]. Our unimodal experiments confirmed previous findings that visual TOJs are less precise than auditory TOJs, and this remained so after training. Based on the cue reliability approach, one would predict that audition should dominate bimodal TOJ performance. However, this prediction cannot be applied to our bimodal experiment because the task did not involve redundant auditory and visual cues. Instead, our task required direct temporal discrimination of sensory sequences. Consequently neither vision nor audition alone provided sufficient information to successfully perform the task. A more valid approach would be to model our bimodal TOJ task as a signed temporal difference between auditory and visual sensory signals. According this view, the greatest improvement in bimodal TOJ performance would arise by reducing the temporal bandwidth of the less temporally precise component, in this case vision, rather than attempting to improve the more precise auditory response. That is, a greater reduction in signed temporal difference arises by improving the bandwidth of the visual modality, relative to the same proportionate improvement in auditory resolution, as this is the component limiting the TOJ discrimination. This proposal is consistent with our data: audiovisual TOJ onset learning improved performance for visual onset, but not for auditory onset (see Figure 2b).

Turning to the unimodal data, there are signifcant differences between the patterns of transfer for vision and audition. The key feature of the visual data was the generalisation of learning. Visual learning transferred strongly across retinal location and feature change, suggesting that visual timing operates after the stage of initial feature coding. Since processing in early visual cortex is highly specific for retinal location and orientation, with cells in V1 exhibiting tight orientation tunings and small retinotopically arranged receptive fields [34], [35], it suggests that the visual clock must be receiving visual inputs after the stage where these features have been extracted. This would be at odds with findings in visual timing which have suggested peripheral visual clocks tied to retinal location [9], although this interpretation has been challenged [36], and it does not square with neurophysiological studies showing duration encoding in non-retinotopic visual areas [37], nor with a variety of evidence pointing to a distributed sub-second timing network involving motor and somatosensory cortices, intrapariental and right parietal areas, and the putamen and cerebellum [1], [11], [38].

The picture emerging from the auditory data is quite the reverse of that implied by the visual data. The striking feature of the auditory data is that there was no transfer at all: not to the visual nor audiovisual tasks, not even within the auditory system to other frequency channels, and not to auditory offset timing within the same frequency channel. The clear implication of such highly specific learning is that the auditory clock operates within frequency channels and, in contrast to visual timing, is therefore likely to be located early in the auditory pathway, possibly even peripheral to primary auditory cortex. As early as the cochlear nucleus, the first significant structure in the ascending auditory pathway after the basilar membrane, the fibres of the auditory nerve are narrowly tuned frequency channels with a bandwidth of about 15% [39], [40]. The cochlear nucleus is a highly organised and laminated structure (especialy the dorsal cochlear nucleus) with sufficient complexity and interneurons to be selectively modified by training [41], [42]. The failure of auditory onset learning to transfer to other frequencies was reported in recent psychophysical study and was similarly interpreted in terms of specific modification of narrowly tuned peripheral auditory filters [24]. In a learning study involving training with speech stimuli, psychophysiological measures showed concommitant modification of the fundamental following response in the rostral brain stem [43]. In sum, there is converging evidence for an early site for specific auditory learning.

Consistent with an early site for auditory onset learning, onset timing is known to be an essential element in auditory perception and it is extracted very early before auditory signals reach the cortex. The reason is that onset timing plays a primary role in the identification of auditory objects. One of the keys to identifying auditory objects is to group their common frequency components based on cues such as common onset and co-modulation of harmonics [44]. If the clocks underlying auditory onset timing were located in the auditory periphery, where they would subserve common onset detection, then the lack of learning transfer between modalities would be expected, as it is difficult to conceive how a visual clock operating at a relatively high level (as the within-modality transfer of visual learning implies) could be influenced by a process that is peripheral and in another sensory modality (i.e., audition). By the same reasoning, it is also unlikely that learning acquired in peripheral auditory processing could benefit a visual timing processes located at a featurally non-specific stage of visual processing.

Apart from implications regarding the level at which visual and auditory learning take place, the observed asymmetry in featural specificity between auditory and visual learning may have a functional and ecological basis. The feature invariant aspect of visual learning might be related to the fact that visual objects, as projected on the retina, frequently change in shape and position. For example, as the observer moves about the environment the viewing perspective on objects changes and this can lead to consequent changes in the shape, size and orientation of the object's retinal projection. Combinations of object motion, eye movements and occlusion may exacerbate this and lead to quite dramatic changes in the projected shape. This can be contrasted with naturally occurring auditory objects, which will tend to maintain their spectral content and object identities despite changes in the listening position. That is, even though changes in listening position may lead to some modulation of the spectral envelope (due to the head-related transfer function: [45]), this is a modulation of intensity in various frequency bands but does not fundamentally alter the frequency content or timing and so does not change cues to object identity such as common onset and co-modulation of harmonics. It may be this relative stability of acoustic spectra of auditory objects that enables feature specific learning to occur in audition, while the inherent variability of visual features may preclude feature-specific visual learning, forcing it to occur at later stages that are less featurally specific. There would be an advantage to doing timing peripherally, where possible, in that shorter response latencies and shorter temporal integration times would lead to less temporal variability.

Finally, one of the clearest results was that onset TOJ learning does not generalise to offset TOJs. Physically, onset and offset judgments contain equivalent temporal information for performing the task. If TOJs were accomplished by employing temporal interval processes, then there should have been transfer from one to the other. However, there was not, suggesting they are separate processes. Other studies have argued that onset and offsets are different. Mossbridge, *et al*. (2008) also found no transfer from auditory onsets to offsets. Although apparently consistent with the notion that onset learning is encoded by different timing mechanisms to those involved in offset judgments, this idea is complicated by the finding that auditory offset learning *does* transfer to onset tasks [46]. Why this asymmetry in the effects of learning should occur is unknown, although it may point to differences in task difficulty associated with onset and offset judgments. Prior to training, the average TOJ threshold across audition, vision, and audiovision for onset was 257 ms, while for offset it was nearly twice as long at 426 ms (p = .008). Anecdotally, subjects commented that offset judgments were far more difficult than onset judgments. Even with quite long offset asynchronies, offset judgments were reported by all observers to require considerably more cognitive effort than onset judgments. This is an odd result as there are data from single-unit neurophysiology studies examining latency times showing that offset responses have a shorter latency and are less temporally variable than onset responses [47], [48], and a similar finding has been reported using visual evoked potentials in response to brief visual presentations [49]. However, our finding does square with studies comparing simple reaction times to stimulus onsets and offsets which have found reaction times to be longer for offset than for onset in both vision [50], [51] and audition [52], [53]. It would appear then that the potential advantage offered by low variability offset responses is not exploited in judgments of temporal order, despite the neurophysiological evidence for temporally precise offset responses. Apart from the conclusion that separate mechanisms underlie timing of onsets and offsets, the data might also reflect the fact that temporal offsets are not as important adaptively to the organism as the temporal discrimination of onsets. Clearly, the organism must respond to events as they happen and timing circuits specialised for onsets would clearly be of greater value, and this seems to be reflected in the vast difference TOJ thresholds for onset and offset, regardless of modality.

Viewed as a whole, the data reported here argue against the simple theoretical dichotomy outlined in the Introduction between separate modality-specific timers on one hand, and a single supramodal timer on the other. There appear to be links between audiovisual timing and visual timing, although based on the evidence from our paradigm auditory timing is governed by a separate process that is highly specific to the trained features and which does not interact with the audiovisual and visual timing network. We have shown that performance in our audio-visual task is limited by the response of the temporally less precise visual mechanism and that the generalisation of audiovisual learning to vision but not audition reflects a strategy that optimises audio-visual performance by increasing the precision of the more sluggish visual mechanism. The lack of generalisation to other auditory frequencies in auditory onset learning combined with the complete generalisation across location and orientation in both visual and audio-visual learning appears to correlate with the variability of visual dimensions in natural viewing compared to the relative stability of auditory spectra. This suggests that TOJ learning may be object-centred rather than viewer-centred, although future research is required to assess the validity of this interpretation. The most robust result without exception is that onset learning does not generalise to offset timing under any circumstances and that offset timing judgments, in all cases, are made with very low acuity relative to onset timing precision. Poor offset precision no doubt reflects the relative unimportance of stimulus offset compared to onsets in decision-making and behavioural response.

## Materials and Methods

### Ethics Statement

Written consent was obtained from each participant prior to the experiments. The experiments were approved by the local ethics committee of the University of Sydney.

### Subjects

A total of eighteen subjects (including the two authors) participated in the experiment. All had normal hearing and normal, or corrected-to-normal visual acuity. All except the authors were naive to the purposes of the study and were unpaid volunteers. The data from one subject in the auditory training group were omitted from the final analyses because their thresholds were extremely deviant (8.9× the average).

### Apparatus

Stimuli were generated using Matlab and the Psychtoolbox [54], [55] on an Apple Macintosh G4 computer running OS 9. Visual stimuli were displayed on a linearised Sony Trinitron monitor (100 Hz vertical refresh, 1024×768 pixel resolution, 8-bit luminance resolution, mean luminance 38 cd/m^2^) and viewed from a distance of 57 cm. Auditory stimuli were played through Beyer Dynamic DT990 headphones.

### Design

A between-subjects design was employed with the 18 observers randomly divided into three training groups, with each group to be trained on temporal onset discrimination in a particular modality: visual onset training, auditory onset training, or audio-visual onset training. The training phase consisted of eight separate days of testing on the temporal order judgment task. The daily training consisted of an 80-trial adaptive staircase to estimate thresholds for onset TOJs, as described below. Subjects were also tested on the day before and the day after the training phase to establish pre-training and post-training performance. The training effect can be quantified by finding the difference between pre- and post-training performance and expressing this as a proportionate change from the pre-training baseline.

The power of the perceptual learning approach is to compare whether the benefit of training generalizes to untrained conditions. This requires measuring performance on a range of “generalization” conditions prior to training and again following training. We tested for generalization between modalities and within modalities. For between modality generalization, a given training group (e.g., auditory) was tested for generality of learning in the other two (untrained) stimulus modalities (i.e., visual and audio-visual). For within modality generalization, the tested modality did not change but the stimuli did (e.g., for vision, the position of the stimulus was changed, or its orientation changed; for audition, the frequency of the tones changed).

### Procedure

On the day prior to the first day of training, TOJ thresholds were measured for stimulus onset and for stimulus offset in the following conditions: Visual (using horizontal grating patches located left and right of fixation); Auditory (using dichotically presented 2000 Hz tones); Audio-visual (using the combined grating patches and dichotic tones). All threshold measurements were completed in separate blocks of trials in a randomised order.

In the training phase, observers made only onset TOJs, and they did so for either visual, auditory or audio-visual stimuli, depending on the training group to which they were allocated. There were 8 days of training, which took place over a range of 8–13 days depending on the subject's availability.

The post-training phase began the day following the final training day. TOJ thresholds were measured again for each pre-training condition (including the training task). A number of additional onset TOJ thresholds were also measured. For visual and audio-visual training groups, two further TOJ thresholds were measured, one in which the visual stimuli were rotated by 45°, and another in which the stimuli remained horizontal but were re-located 10° above and below (rather than left and right) of fixation. For the auditory training group, an additional TOJ threshold was measured using frequencies half an octave higher than in the training phase.

### Measurement of TOJ thresholds for onset and offset

For onset thresholds, subjects made a laterality judgment, indicating on which side of fixation the stimulus (whether visual, auditory, or audio-visual) occurred first, with the presentation order randomised over trials. The onset asynchrony was varied to find the onset TOJ threshold, and the stimuli were extinguished synchronously after 2 seconds. For offset thresholds, the stimuli were presented with synchronous onset, but were extinguished asynchronously after about 2 seconds. Subjects indicated which side was extinguished first and the offset asynchrony was varied to find the offset TOJ threshold. For audio-visual trials, the visual stimuli were synchronized with each other, as were the auditory stimuli, and the asynchrony was defined by the difference between the auditory and visual modalities.

An adaptive staircase procedure [56] was used to vary the stimulus onset (or offset) asynchrony to find the TOJ threshold. Incorrect judgments increased the stimulus asynchrony and correct judgments decreased it. The data from two randomly interleaved staircases of 40 trials each were pooled and fitted with a cumulative Gaussian psychometric function, the mean of which defined the TOJ threshold (the asynchrony at which performance was 75% correct). Each threshold measurement took approximately five minutes.

### Stimulus details

#### Visual stimuli

**Figure 3:**
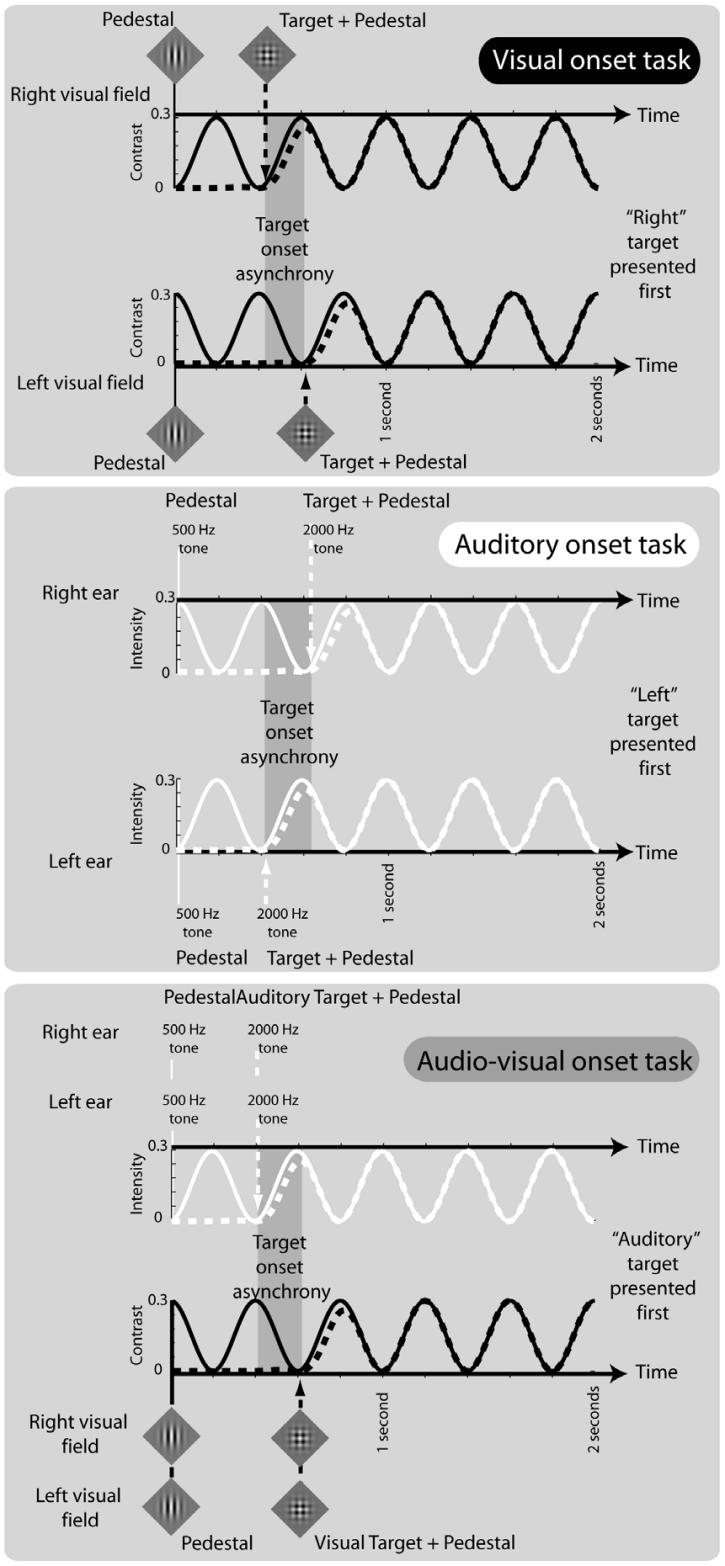
Temporal structure of stimuli in each of the three training conditions. Top: visual onset training; middle: auditory onset training; bottom: audio-visual onset training. Visual and auditory stimuli are represented as black and white curves respectively. Each stimulus condition is composed of two targets: left vs. right of fixation (visual onset condition); to left vs right ears (auditory onset condition); and visually vs. auditorily (audio-visual onset condition). Within each trial, target increment onsets (dotted curves) are delayed with respect to each other by “target onset asynchrony” (vertical shaded region) and are linearly summed with a pedestal presented throughout the trial (solid curves). As described in the Methods, each target/pedestal combination is amplitude modulated at 2.5 Hz throughout the trial, with a temporal phase difference of 180° applied to each.

There were two components to the visual stimuli: a *pedestal* of vertical grating that was present throughout the 2-second stimulus duration (one on each side of the display), and a horizontal *test* grating which appeared after the pedestal (randomly between 300–500 ms) and whose onset asynchrony relative to the test stimulus at the other location was varied (see Figure 3a). The grating patches were Gabor patterns, meaning they were composed of a sine-wave carrier grating (spatial frequency of 3.5 cyc/deg) windowed by a Gaussian envelope (standard deviation of 60 pixels).

The reason for the pedestal arrangement was that strong apparent motion resulted if test gratings alone were presented asynchronously, giving a strong cue that made the TOJ task easy. As our interest concerned the limit for judging the order of discrete events rather than the temporal resolution of apparent motion, we took several steps to overcome this. First, we used a synchronously presented, high-contrast pedestal prior to presenting the test gratings, which was very effective at masking the apparent motion cue between the asynchronous test gratings. To further reduce apparent motion, we spread the visual stimuli well apart spatially (±10° either side of a small central fixation cross) and the onsets and offsets of the target were temporally smoothed using a cumulative Gaussian ramp with a standard deviation of 10 ms. As a final precaution against this cue, the combined target and pedestal gratings were amplitude modulated with a 2.5 Hz sine wave that modulated the total contrast between 0 and 30%. Perceptually, this resulted in the appearance of pulsating vertical pedestal gratings either side of fixation, followed by the asynchronous onset of orthogonal (and pulsating) gratings. To help keep the left and right sides of the display independent, the temporal modulations on each side were out of phase by 180° (see Figure 3a).

The visual “offset” condition was identical to the “onset” condition described above except that the Gaussian onset ramps for the target were reversed temporally. This meant that the target and pedestal were synchronously presented at the beginning of the trial, with the target gratings offsetting asynchronously towards the end of the trial (randomly within 300–500 ms of the end). The horizontal spatial configuration of the stimuli (±10° either side of fixation) was used in all conditions except the “visual onset location change” condition in which the test patches were located 10° above and below fixation. Finally, the vertical and horizontal orientations of the pedestal and grating was using in all conditions except the “visual onset +45°” condition in which the orientations of both pedestal and target were rotated by 45°.

#### Auditory stimuli

For consistency with the visual condition, the same two-component arrangement (pedestal and test stimuli) was used for the auditory stimuli, and all aspects of stimulus timing were the same. The pedestal was a 500 Hz tone, and the target was a 2000 Hz tone. Frequency intensities were equalised perceptually for each subject by first measuring detection thresholds for each frequency and then adding a constant intensity increment of 60 dB to each. The pedestal tone was presented dichotically and was present for the entire stimulus period, with the target presented asynchronously to each ear 300 to 500 ms later (see Figure 3b). As for the visual stimuli, a 2.5 Hz amplitude modulation was applied to the combined target and pedestal signal (modulating between 0 and 60 dB), with each ear's modulation 180° out-of-phase with the other. The auditory “offset” condition was identical spatially to the “onset” condition except that target temporal cosine ramps were reversed.

#### Audio-visual stimuli

The bimodal stimuli were composed of the same auditory and visual components described above. The asynchrony in this case was between the targets components of the auditory and visual stimuli. That is, within each modality, the targets were synchronous, but between modalities they were asynchronous. The 2.5 Hz amplitude modulation of the stimuli was in phase within modalities but was 180° out of phase between modalities (see Figure 3c).

## References

[1] Mauk MD, Buonomano DV (2004). The neural basis of temporal processing.. Annu Rev Neurosci.

[2] Michon JA, Michon JA, Jackson JL (1985). The complete time experiencer.. Time, Mind and Behavior.

[3] Edwards CJ, Alder TB, Rose GJ (2002). Auditory midbrain neurons that count.. Nat Neurosci.

[4] Schirmer A (2004). Timing speech: a review of lesion and neuroimaging findings.. Brain Res Cogn Brain Res.

[5] Alais D, Newell FN, Mamassian P (2010). Multisensory processing in review: from physiology to behaviour.. Seeing Perceiving.

[6] Stein BE, Stanford TR (2008). Multisensory integration: current issues from the perspective of the single neuron.. Nat Rev Neurosci.

[7] Allan LG (1979). The perception of time.. Percept Psychophys.

[8] Nagarajan SS, Blake DT, Wright BA, Byl N, Merzenich MM (1998). Practice-related improvements in somatosensory interval discrimination are temporally specific but generalize across skin location, hemisphere, and modality.. J Neurosci.

[9] Johnston A, Arnold DH, Nishida S (2006). Spatially localized distortions of event time.. Curr Biol.

[10] Morgan MJ, Giora E, Solomon JA (2008). A single “stopwatch” for duration estimation, a single “ruler” for size.. J Vis.

[11] Buhusi CV, Meck WH (2005). What makes us tick? Functional and neural mechanisms of interval timing.. Nat Rev Neurosci.

[12] Divenyi PL, Sachs RM (1978). Discrimination of time intervals bounded by tone bursts.. Percept Psychophys.

[13] Rousseau R, Poirier J, Lemyre L (1983). Duration discrimination of empty time intervals marked by intermodal pulses.. Percept Psychophys.

[14] Getty DJ (1975). Discrimination of short temporal intervals: a comparison of two models.. Percept Psychophys.

[15] Westheimer G (1999). Discrimination of short time intervals by the human observer.. Exp Brain Res.

[16] Grondin S, Meilleur-Wells G, Ouellette C, Macar F (1998). Sensory effects on judgments of short time-intervals.. Psychol Res.

[17] Rammsayer TH, Vogel WH (1992). Pharmacologic properties of the internal clock underlying time perception in humans.. Neuropsychobiology.

[18] Harrington DL, Haaland KY, Hermanowicz N (1998). Temporal processing in the basal ganglia.. Neuropsychology.

[19] Fahle M, Poggio T (2002). Perceptual Learning..

[20] Sowden PT, Rose D, Davies IR (2002). Perceptual learning of luminance contrast detection: specific for spatial frequency and retinal location but not orientation.. Vision Res.

[21] Folta K (2003). Neural fine tuning during Vernier acuity training?. Vision Res.

[22] Karmarkar UR, Buonomano DV (2003). Temporal specificity of perceptual learning in an auditory discrimination task.. Learn Mem.

[23] Wright BA, Buonomano DV, Mahncke HW, Merzenich MM (1997). Learning and generalization of auditory temporal-interval discrimination in humans.. J Neurosci.

[24] Mossbridge JA, Fitzgerald MB, O'Connor ES, Wright BA (2006). Perceptual-learning evidence for separate processing of asynchrony and order tasks.. J Neurosci.

[25] Seitz AR, Kim R, Shams L (2006). Sound facilitates visual learning.. Curr Biol.

[26] Kim RS, Seitz AR, Shams L (2008). Benefits of stimulus congruency for multisensory facilitation of visual learning.. PLoS One.

[27] Majewska AK, Sur M (2006). Plasticity and specificity of cortical processing networks.. Trends Neurosci.

[28] Pascual-Leone A, Amedi A, Fregni F, Merabet LB (2005). The plastic human brain cortex.. Annu Rev Neurosci.

[29] Bavelier D, Neville HJ (2002). Cross-modal plasticity: where and how?. Nat Rev Neurosci.

[30] Virsu V, Oksanen-Hennah H, Vedenpää A, Jaatinen P, Lahti-Nuuttila P (2008). Simultaneity learning in vision, audition, tactile sense and their cross-modal combinations.. Exp Brain Res.

[31] Burr D, Alais D (2006). Combining visual and auditory information.. Prog Brain Res.

[32] Alais D, Burr D (2004). The ventriloquist effect results from near-optimal bimodal integration.. Curr Biol.

[33] Ernst MO, Banks MS (2002). Humans integrate visual and haptic information in a statistically optimal fashion.. Nature.

[34] Grill-Spector K, Malach R (2004). The human visual cortex.. Annu Rev Neurosci.

[35] Lennie P (1998). Single units and visual cortical organization.. Perception.

[36] Burr D, Tozzi A, Morrone MC (2007). Neural mechanisms for timing visual events are spatially selective in real-world coordinates.. Nat Neurosci.

[37] Leon MI, Shadlen MN (2003). Representation of time by neurons in the posterior parietal cortex of the macaque.. Neuron.

[38] Coull JT, Nazarian B, Vidal F (2008). Timing, Storage, and Comparison of Stimulus Duration Engage Discrete Anatomical Components of a Perceptual Timing Network.. J Cogn Neurosci.

[39] Moore BCJ (1986). Frequency Selectivity in Hearing..

[40] Patterson RD (1976). Auditory filter shapes derived with noise stimuli.. J Acoust Soc Am.

[41] Oertel D (1999). The role of timing in the brain stem auditory nuclei of vertebrates.. Annu Rev Physiol.

[42] Fujino K, Oertel D (2003). Bidirectional synaptic plasticity in the cerebellum-like mammalian dorsal cochlear nucleus.. Proc Natl Acad Sci U S A.

[43] Song JH, Skoe E, Wong PC, Kraus N (2008). Plasticity in the Adult Human Auditory Brainstem following Short-term Linguistic Training.. J Cogn Neurosci.

[44] Bregman AS (1990). Auditory Scene Analysis: The Perceptual Organization of sound..

[45] Carlile S (1996). Virtual auditory space: Generation and applications..

[46] Mossbridge JA, Scissors BN, Wright BA (2008). Learning and generalization on asynchrony and order tasks at sound offset: implications for underlying neural circuitry.. Learn Mem.

[47] Bair W (2004). No doubt about offset latency.. Vis Neurosci.

[48] Bair W, Cavanaugh JR, Smith MA, Movshon JA (2002). The timing of response onset and offset in macaque visual neurons.. J Neurosci.

[49] Serviere J, Miceli D, Galifret Y (1977). Electrophysiological correlates of the visual perception of “instantaneous” and “durable”.. Vision Res.

[50] Efron R (1970). Effect of stimulus duration on perceptual onset and offset latencies.. Percept Psychophys.

[51] Parker DM (1980). Simple reaction times to the onset, offset, and contrast reversal of sinusoidal grating stimuli.. Percept Psychophys.

[52] Rammsayer TH (1998). Sex-related differences in reaction times to onset and offset of auditory stimuli.. Psychologische Beiträge.

[53] King B, Wood C, Faulkner D (2007). Sensitivity to auditory and visual stimuli during early reading development.. Journal of Research in Reading.

[54] Brainard DH (1997). The Psychophysics Toolbox.. Spat Vis.

[55] Pelli DG (1997). The VideoToolbox software for visual psychophysics: transforming numbers into movies.. Spat Vis.

[56] Watson AB, Pelli DG (1983). QUEST: a Bayesian adaptive psychometric method.. Percept Psychophys.

